# Supplementary material for: Low-Cost Microfabrication Tool Box
Source: Micromachines (Basel). 2020 Jan 25;11(2):135. doi: 10.3390/mi11020135 (PMC7074766; doi:10.3390/mi11020135)
Supplement: Supplementary file 1 [file micromachines-11-00135-s001.zip › Supplementary Materials/GCode_MicroMilling.docx]

G76

G90

M10 O6.1

G0 X50. Y12.5

G0 Z10.

G0 Z5.

G1 Z2.5 F10.

G1 Z-0.1

G1 X46.

G3 X45. Y11.5 I0. J-1.

G1 Y8.5

G2 X44. Y7.5 I-1. J0.

G1 X43.5

G2 X42.5 Y8.5 I0. J1.

G1 Y16.5

G3 X41.5 Y17.5 I-1. J0.

G1 X41.

G3 X40. Y16.5 I0. J-1.

G1 Y8.5

G2 X39. Y7.5 I-1. J0.

G1 X38.5

G2 X37.5 Y8.5 I0. J1.

G1 Y16.5

G3 X36.5 Y17.5 I-1. J0.

G1 X36.

G3 X35. Y16.5 I0. J-1.

G1 Y8.5

G2 X34. Y7.5 I-1. J0.

G1 X33.5

G2 X32.5 Y8.5 I0. J1.

G1 Y16.5

G3 X31.5 Y17.5 I-1. J0.

G1 X31.

G3 X30. Y16.5 I0. J-1.

G1 Y13.5

G2 X29. Y12.5 I-1. J0.

G1 X26.1

G3 X25. Y11.4 I0. J-1.1

G1 Y7.5

G1 Y11.4

G2 X26.1 Y12.5 I1.1 J0.

G1 X29.

G3 X30. Y13.5 I0. J1.

G1 Y16.5

G2 X31. Y17.5 I1. J0.

G1 X31.5

G2 X32.5 Y16.5 I0. J-1.

G1 Y8.5

G3 X33.5 Y7.5 I1. J0.

G1 X34.

G3 X35. Y8.5 I0. J1.

G1 Y16.5

G2 X36. Y17.5 I1. J0.

G1 X36.5

G2 X37.5 Y16.5 I0. J-1.

G1 Y8.5

G3 X38.5 Y7.5 I1. J0.

G1 X39.

G3 X40. Y8.5 I0. J1.

G1 Y16.5

G2 X41. Y17.5 I1. J0.

G1 X41.5

G2 X42.5 Y16.5 I0. J-1.

G1 Y8.5

G3 X43.5 Y7.5 I1. J0.

G1 X44.

G3 X45. Y8.5 I0. J1.

G1 Y11.5

G2 X46. Y12.5 I1. J0.

G1 X50.

G0 Z10.

M10 O6.1

G0 Z15.

G0 X25. Y17.5

G0 Z5.

G1 Z2.5 F10.

G1 Z-0.1

G1 Y13.6

G3 X26.1 Y12.5 I1.1 J0.

G1 X27.5

G1 X26.1

G2 X25. Y13.6 I0. J1.1

G1 Y17.5

G0 Z15.

M10 O6.0

G77

G99
